# Cytologic and Cytochemical Characterization of Blue-Green Neutrophilic Inclusions in a Dog with Suspected Transfusion-Associated Hemolysis

**DOI:** 10.3390/vetsci13090985

**Published:** 2026-09-18

**Authors:** Hyun-Jeong Hwang, Myung Hyun Choi, Hyo-Sung Kim, Sun Hee Do

**Affiliations:** Department of Veterinary Clinical Pathology, College of Veterinary Medicine, Konkuk University, Seoul 05029, Republic of Korea; ibbng@naver.com (H.-J.H.); robin3384@naver.com (M.H.C.); w6373515@naver.com (H.-S.K.)

**Keywords:** blue-green leukocyte inclusions, neutrophils, suspected transfusion-associated hemolysis, Cytochemistry, lipofuscin-like material, dog

## Abstract

An 11-year-old castrated male Maltese dog presented with acute lethargy and non-regenerative anemia. Despite immunosuppressive therapy and an initial packed red blood cell (pRBC) transfusion, hematocrit levels progressively declined after transient improvement. A second transfusion was administered following compatible crossmatch testing. Within 24 h of the second transfusion, a transfusion-associated hemolytic episode was suspected due to a rapid hematocrit decline, pigmenturia, hyperbilirubinemia, and autoagglutination. Peripheral blood smear revealed neutrophils containing coarse blue-green cytoplasmic inclusions. Special staining and autofluorescence microscopy supported lipid-rich, acid-fast, and autofluorescent properties of the inclusions. Prussian blue staining showed iron-positive granular material in the hemolytic sample, but the intraneutrophilic inclusions did not show distinct Prussian blue reactivity, making hemosiderin-rich sideroleukocytes unlikely to fully explain the findings. Similar inclusions have recently been characterized in dogs, mainly in association with severe hepatocellular injury, pancreatitis, and poor short-term outcomes; however, their occurrence during acute clinical deterioration associated with suspected post-transfusion hemolysis has not been specifically documented. The dog died three days after inclusion detection. This case expands the clinical settings in which blue-green leukocyte cytoplasmic inclusions may be encountered in dogs.

## Simple Summary

This report describes unusual blue-green cytoplasmic inclusions in neutrophils from a critically ill dog during acute clinical deterioration following a packed red blood cell transfusion. A suspected transfusion-associated hemolytic episode was considered based on the rapid decline in hematocrit, pigmenturia, marked hyperbilirubinemia, and autoagglutination. Cytochemical staining and autofluorescence showed characteristics compatible with previously described blue-green leukocyte inclusions and lipofuscin-like material. Prussian blue staining demonstrated iron-containing material in the blood smear, whereas representative blue-green intraneutrophilic inclusions lacked distinct Prussian blue reactivity, making a purely hemosiderin-based explanation less likely. Because blood smears obtained immediately before the event were unavailable and concurrent hepatocellular injury could not be excluded, a causal relationship between transfusion, hemolysis, and inclusion formation cannot be established. This case expands the clinical context in which blue-green leukocyte inclusions may be encountered in dogs.

## 1. Introduction

Blue-green leukocyte cytoplasmic inclusions have been described in critically ill human patients and have variously been referred to as “critical green inclusions” or “green neutrophilic inclusions”. First identified in 2009, these inclusions appear as blue-green amorphous or granular cytoplasmic bodies, typically in terminally ill patients with hepatic failure, sepsis, or multiorgan dysfunction. Their appearance is frequently associated with poor outcomes and mortality within days [1,2].

Although these inclusions were initially recognized in human clinical pathology, similar blue-green leukocyte inclusions have recently been characterized in dogs, where they were associated mainly with severe hepatocellular injury, pancreatitis, systemic inflammation, and poor short-term outcomes [3]. Here, we describe and cytochemically characterize blue-green neutrophilic cytoplasmic inclusions detected during acute clinical deterioration temporally associated with suspected transfusion-associated hemolysis. The present case is intended to expand the clinical context in which these inclusions may be encountered rather than to establish hemolysis or transfusion as their cause.

## 2. Case Description

**Figure 1:**
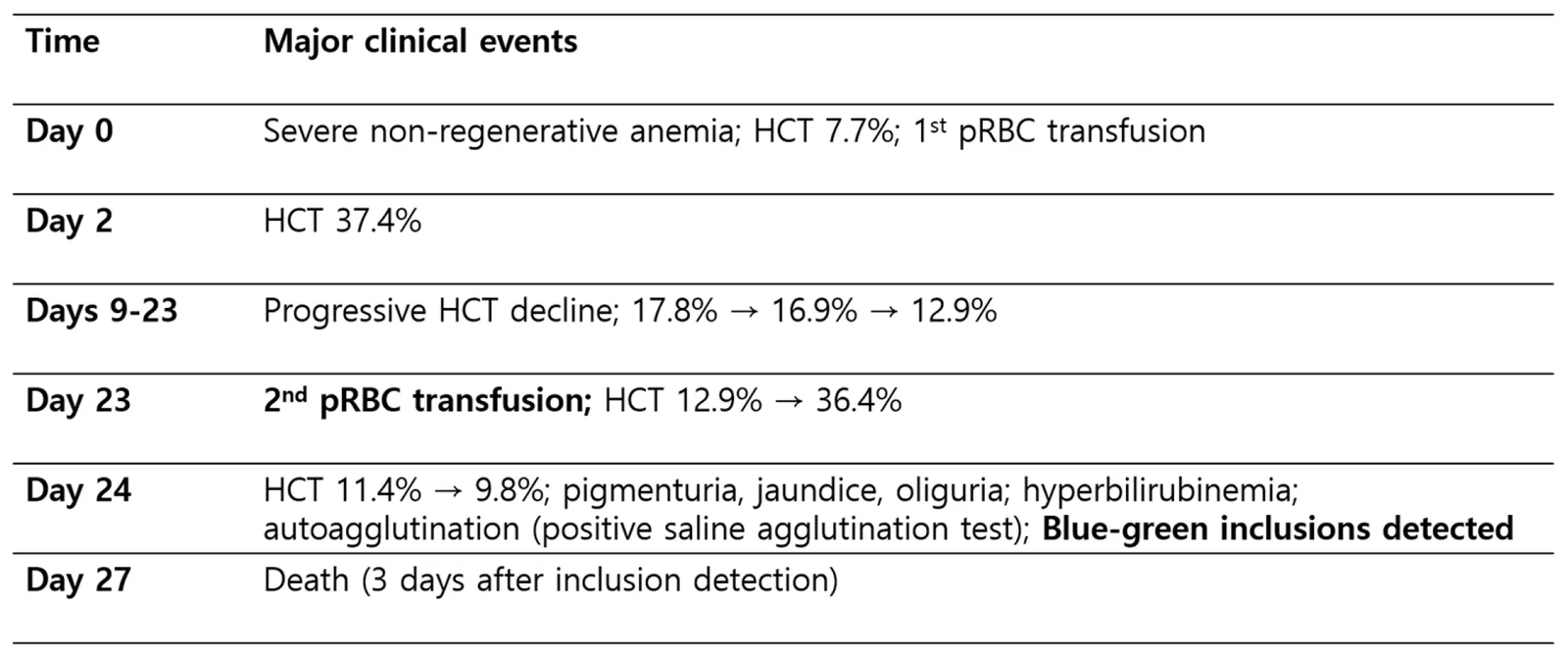
Timeline of the major clinical events, packed red blood cell transfusions, hematologic changes, detection of blue-green neutrophilic cytoplasmic inclusions, and clinical outcome. HCT, hematocrit; pRBC, packed red blood cells.

An 11-year-old castrated male Maltese dog was presented with acute lethargy and anorexia lasting three days. Prior fluid therapy at a local clinic resulted in a 20% weight gain and mild pulmonary edema, interstitial and bronchial signs in multiple lung lobes with pleural effusion, presumed secondary to overhydration. On presentation, physical exam revealed a weak pulse but no signs of dehydration. Complete blood count revealed severe non-regenerative anemia with a low hematocrit (HCT) of 7.7%, confirmed by reticulocyte staining with new methylene blue (Table 1, Figure 1). Poikilocytosis with spherocytes, acanthocytes, and ghost cells was present. Coagulation testing showed increased D-dimer and thrombocytosis. Autoagglutination was not observed initially. Although the findings were not typical of classic regenerative immune-mediated hemolytic anemia (IMHA) at presentation, an immune-mediated anemia of undetermined subtype, including non-regenerative immune-mediated anemia such as precursor-targeted immune-mediated anemia, could not be excluded.

Following crossmatch testing, the first pRBC transfusion was administered. At the time of the first transfusion, the dog weighed approximately 2.3 kg. Crossmatching was negative, and 40 mL of pRBC diluted with 10 mL of sterile saline was administered. The medical record also mentioned an additional 25 mL transfusion if hemorrhage occurred; however, administration of this additional volume could not be confirmed. The exact infusion rate and duration of the first transfusion were unavailable from the retrospective medical record. The patient temporarily improved: HCT increased to 37.4%, and thoracic radiographic findings improved. Abdominal ultrasonography did not reveal an obvious source of intra-abdominal hemorrhage, although occult blood loss could not be completely excluded. Following the initiation of immunosuppressive therapy, the patient exhibited improved appetite and vitality alongside a decrease in spherocytes. However, because the anemia persisted, as indicated by a low corrected reticulocyte index and minimal reticulocytosis, immunosuppressive therapy with prednisolone and mycophenolate mofetil was continued. Concurrent antithrombotic therapy with clopidogrel and rivaroxaban was also maintained. Although abdominal ultrasonography did not identify an overt source of intra-abdominal hemorrhage, occult blood loss during antithrombotic therapy could not be completely excluded as a contributor to the progressive anemia. PCR tests for vector-borne pathogens (*Babesia* spp., *Anaplasma* spp., *Ehrlichia* spp., *Rickettsia* spp., *Hepatozoon* spp., *Leishmania* spp., *Neorickettsia risticii*, *Bartonella* spp., *Mycoplasma haemocanis*, *Mycoplasma haematoparvum*) were negative. Over the following 23 days, HCT gradually declined below 15%, and a second transfusion was indicated. At the time of the second transfusion on Day 23, the dog weighed 2.065 kg. Crossmatching using an Alvedia kit was negative. Based on a target PCV of approximately 35%, an initial pRBC volume of 50 mL (24.2 mL/kg) was calculated and diluted with 30 mL of sterile saline. The transfusion was initiated at approximately 12:15 at 2.5 mL/h, with the infusion rate progressively increased to 22 mL/h under serial monitoring of vital signs. An additional 30 mL of pRBC was subsequently administered, resulting in a total documented pRBC volume of 80 mL (approximately 38.7 mL/kg). The transfusion was completed at approximately 16:36. HCT transiently increased from 12.9% to 36.4%. The first documented body temperature after initiation of the transfusion was 38.2 °C, and the temperature subsequently increased to a documented peak of 39.7 °C. Cooling was initiated at approximately 39.5 °C, after which the temperature decreased to 38.9 °C. No marked respiratory or cardiovascular instability was documented during the transfusion. Blood-type information for the recipient and donor was unavailable in the retrospective medical record.

A transfusion-associated hemolytic episode was suspected based on the temporal association with the second pRBC transfusion, rapid hematocrit decline, pigmenturia suggestive of hemoglobinuria, hyperbilirubinemia, and autoagglutination. Blood work revealed oliguria with progressive azotemia, compatible with renal dysfunction, with a blood urea nitrogen (BUN) of 65 mg/dL, and creatinine of 1.0 mg/dL. There was also marked hyperbilirubinemia (total bilirubin, 17.9 mg/dL), increased C-reactive protein (CRP) level (8.6 mg/dL), and hyperlactatemia (lactate, 4.02 mmol/L) (Table 2, Figure 1). The markedly increased mean corpuscular hemoglobin concentration (MCHC) values after clinical deterioration were considered artifactual and most likely reflected analytical interference associated with severe hemolysis and icterus rather than true erythrocyte hyperchromasia (Table 1). Within 24 h after the second transfusion, HCT rapidly decreased to 11.4% and subsequently to 9.8%, and autoagglutination was confirmed by a saline agglutination test.

**Figure 2:**
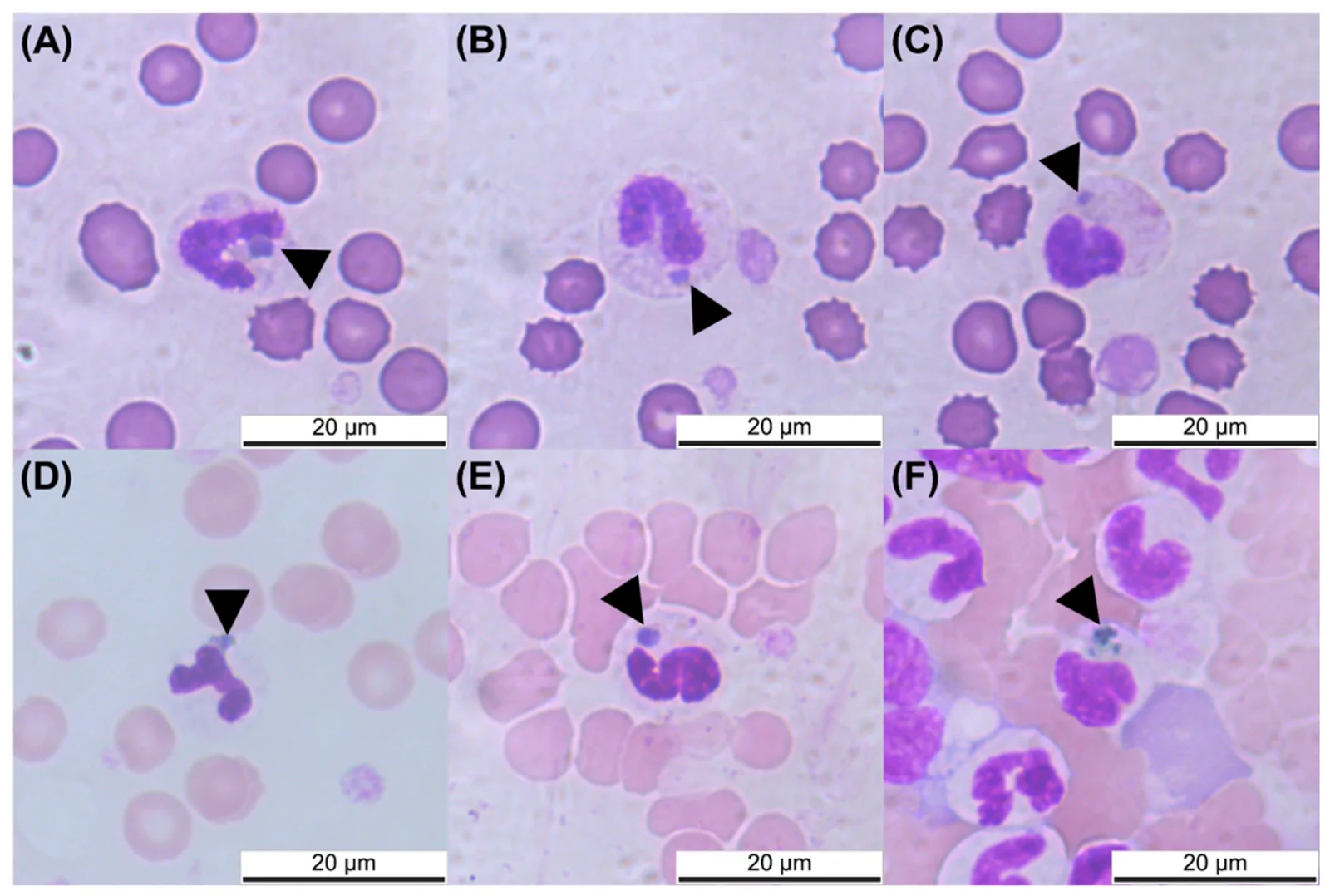
Representative images of cytoplasmic coarse, blue-green intraneutrophilic inclusions (arrowheads) in peripheral blood smear. (**A**–**C**) Diff-Quik staining; (**D**–**F**) Wright-Giemsa staining. All images were photographed at 1000× magnification with immersion oil.

**Figure 3:**
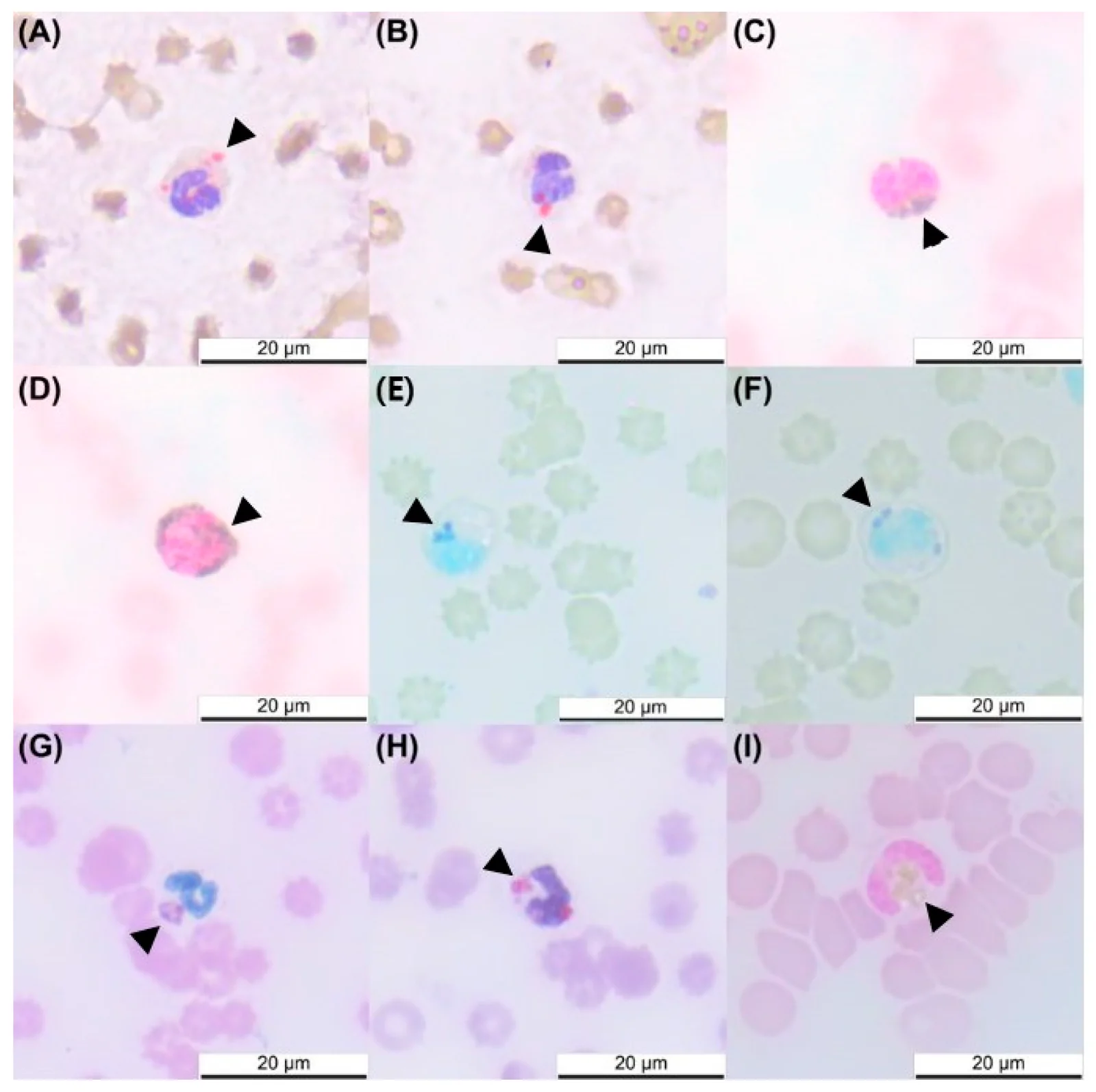
Cytoplasmic inclusions visualized using various special stains. Arrowheads indicate representative intraneutrophilic inclusions in each panel. (**A**,**B**) Oil Red O staining, counterstained with hematoxylin; (**C**,**D**) Sudan Black B staining, counterstained with nuclear fast red; (**E**,**F**) Nile Blue staining; (**G**,**H**) Long Ziehl-Neelsen staining, counterstained with new methylene blue (**G**) and hematoxylin (**H**); (**I**) Prussian blue staining showing tan intraneutrophilic granules without distinct Prussian blue reactivity, despite the presence of Prussian blue-positive granular material in the smear. All images were photographed at 1000× magnification with immersion oil. The panels represent independently selected representative cells/fields from separately stained blood smears; they do not depict serial staining of the same cell.

**Figure 4:**
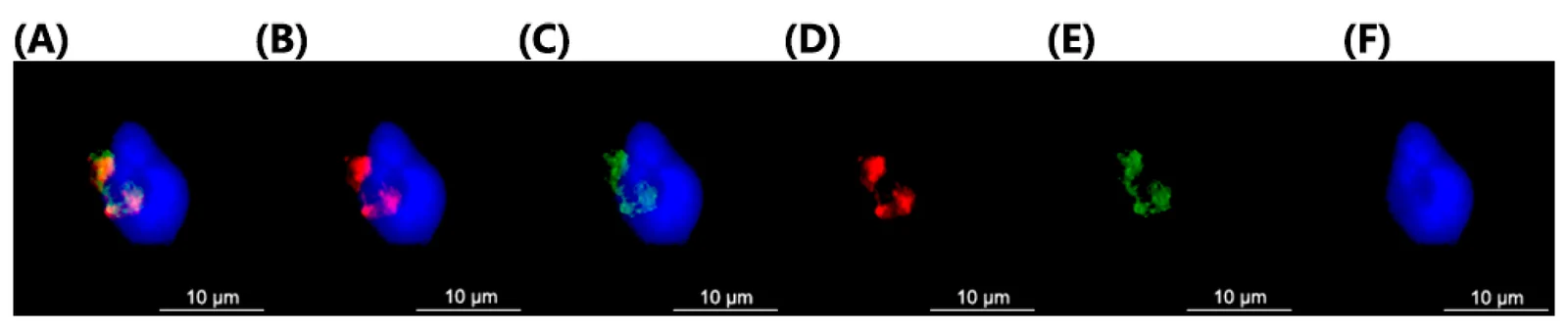
Autofluorescence of blue-green neutrophilic cytoplasmic inclusions. Peripheral blood smears were counterstained with DAPI for nuclear visualization, and inclusion-associated autofluorescence was evaluated using L5 (green) and N2.1 (red) fluorescence filter cubes. (**A**) Merged images; (**B**) red fluorescence/DAPI merged; (**C**) green fluorescence/DAPI merged; (**D**) red fluorescence (N2.1); (**E**) green fluorescence (L5); (**F**) DAPI nuclear fluorescence (**D**). Images were acquired using a Leica DM2500 fluorescence microscope equipped with an N PLAN 100×/1.25 oil-immersion objective.

Air-dried peripheral blood smears were used for cytochemical characterization of the inclusions. Oil Red O staining was performed using an Oil Red O Stain kit (ab150678; Abcam, Cambridge, UK), and Sudan Black B staining was performed using a Sudan Black B Staining System (380B; Sigma-Aldrich, St. Louis, MO, USA), according to the manufacturers’ instructions. Prussian blue staining was performed using an Iron Stain Kit (ab150674; Abcam) according to the manufacturer’s instructions. Nile Blue staining was performed using Nile Blue A (N0766; Sigma-Aldrich) following a standard staining procedure; blue to navy-blue coloration of the morphologically identifiable intraneutrophilic inclusions was interpreted as positive reactivity. Long Ziehl-Neelsen staining was performed using Ziehl-Neelsen carbol-fuchsin solution (109215; Merck, Darmstadt, Germany) with a modified staining procedure. Filtered carbol fuchsin was applied at 60 °C for 3 h, followed by differentiation in 1% acid alcohol and counterstaining with aqueous methylene blue or hematoxylin. Staining reactions were evaluated according to the characteristic color of the reaction product and its localization within the morphologically identifiable intraneutrophilic inclusions. A peripheral blood smear revealed numerous neutrophils containing coarse green-blue cytoplasmic inclusions (Figure 2). Special stains were performed to characterize the inclusions, including Oil Red O, Sudan Black B, Nile Blue, and Long Ziehl-Neelsen staining; the latter was counterstained with either new methylene blue or hematoxylin to assess the acid-fast properties of the inclusions. Prussian blue staining was additionally performed to evaluate iron-containing pigment. In the hemolytic sample, Prussian blue-positive granular material was observed, consistent with the presence of iron-containing pigment in the setting of severe hemolysis. However, some intraneutrophilic tan granules corresponding to the blue-green inclusions did not show distinct Prussian blue reactivity. The absence of distinct Prussian blue reactivity in representative blue-green intraneutrophilic inclusions reduced the likelihood that these inclusions were composed of predominantly hemosiderin; however, this finding did not establish their alternative chemical identity (Figure 3). Autofluorescence was evaluated on separate air-dried peripheral blood smears that had not undergone cytochemical staining or decolorization. The smears were counterstained with DAPI for nuclear visualization, without application of other fluorescent dyes. Fluorescence was evaluated using a Leica DM2500 fluorescence microscope equipped with a DFC 420 C camera and an N PLAN 100×/1.25 oil-immersion objective (Leica Microsystems, Wetzlar, Germany). Images were acquired using LAS X software (version 2.0.0.14332) with D (blue), L5 (green), and N2.1 (red) fluorescence filter cubes. The blue-green intraneutrophilic inclusions exhibited autofluorescence in the green and red channels (Figure 4). Leukocytes without inclusions and adjacent background areas on the same slide served as internal negative comparisons and showed no comparable autofluorescence in these channels. Despite supportive care, the dog died three days after the inclusions were detected.

Morphologically similar inclusions, historically referred to as “death crystals” or “critical green inclusions” have been reported in critically ill human patients with severe hepatocellular injury or multiorgan dysfunction [4,5]. In dogs, comparable inclusions have also been reported, most notably in association with hepatocellular injury [3].

## 3. Discussion

The cause of the initial severe non-regenerative anemia could not be definitively established. Although an immune-mediated process was suspected based on the presence of spherocytes and ghost cells and the subsequent decrease in spherocytes during immunosuppressive treatment, the lack of an adequate regenerative response and absence of bone marrow examination precluded definitive classification. Moreover, the progressive decline in HCT following the first transfusion could have reflected persistence or progression of the underlying anemia, immune-mediated or non-immune-mediated erythrocyte destruction, a delayed transfusion-related process, or occult blood loss. These possibilities could not be distinguished retrospectively and should be considered separately from the acute clinical deterioration that occurred after the second transfusion. The respiratory abnormalities noted before the first transfusion were more consistent with fluid overload from prior treatment than with transfusion-related acute lung injury. According to the Association of Veterinary Hematology and Transfusion Medicine (AVHTM) Transfusion Reaction Small Animal Consensus Statement (TRACS), transfusion reactions occurring within 24 h of blood product administration are classified as acute transfusion reactions [6]. In the present case, the temporal association between the second transfusion and the subsequent rapid decline in HCT, pigmenturia, hyperbilirubinemia, and autoagglutination was clinically compatible with a suspected acute hemolytic transfusion reaction. However, definitive classification of this event was not possible because post-reaction compatibility testing, direct antiglobulin testing, plasma-free hemoglobin measurement, formal post-reaction urinalysis, and detailed donor/unit information were unavailable.

Cytoplasmic blue-green inclusions observed in neutrophils and monocytes, often referred to as “critical green inclusions” or “death crystal-like” inclusions, have been increasingly recognized in human clinical pathology. Since their initial description, these inclusions have been reported in critically ill patients with severe hepatic disease, sepsis, multiorgan dysfunction, and terminal clinical deterioration [7,8,9]. Although their presence has often been associated with poor short-term outcomes, subsequent reports have shown that they are not uniformly fatal and should not be interpreted as definitive predictors of death [10,11]. Therefore, these inclusions are best regarded as cytologic indicators of severe systemic illness rather than independent prognostic markers. In the present case, inclusion detection coincided with rapid clinical deterioration and death within three days; however, a direct causal or independent prognostic relationship could not be established.

Blue-green leukocyte inclusions have recently been characterized in dogs and were reported to have lipofuscin-like staining and autofluorescent properties [3]. In that canine series, affected dogs commonly had hepatocellular injury or pancreatitis, and many experienced poor short-term outcomes. In contrast, neutrophilic inclusions were detected in the present dog during acute clinical deterioration after a suspected transfusion-associated hemolytic episode, accompanied by marked hyperbilirubinemia, severe hemolysis, pigmenturia suggestive of hemoglobinuria, progressive azotemia with oliguria suggestive of renal dysfunction, hyperlactatemia, and systemic inflammation. Because hepatocellular enzyme activities were not measured at the time inclusions were first detected, concurrent hepatocellular injury, a major known association of these inclusions, could not be excluded. The available data therefore cannot distinguish whether the inclusions were associated with hepatocellular injury, suspected hemolysis, systemic inflammation/critical illness, or a combination of these processes. A contribution from hemolysis-associated hypoxic or oxidative injury remains a possible but untested hypothesis. The inclusions were visible on both Diff-Quik and Wright-Giemsa stains and showed positive staining with Oil Red O, Sudan Black B, and Long Ziehl-Neelsen, together with green-red autofluorescence. Because sideroleukocytes are an important differential diagnosis in the setting of severe hemolysis, Prussian blue staining was performed to evaluate iron-containing pigment. Prussian blue-positive granular material was present in the hemolytic smear; however, the tan intraneutrophilic granules corresponding to some of the observed inclusions lacked distinct Prussian blue reactivity. Therefore, although iron-containing pigment may have been present in the sample, hemosiderin-rich sideroleukocytes were unlikely to be the sole explanation for the blue-green neutrophilic inclusions. Taken together with the lipid-positive, acid-fast, and autofluorescent properties, the combined cytochemical and autofluorescence findings were compatible with lipofuscin-like material. These staining characteristics are not specific for lipofuscin and do not establish a definitive chemical identity.

Lipofuscin is a non-degradable byproduct of lipid peroxidation and protein oxidation, typically accumulating in long-lived or stressed cells, particularly within hepatocytes and cardiomyocytes. The presence of lipofuscin-like material in circulating leukocytes may reflect systemic oxidative stress or widespread tissue injury. In human reports, such inclusions have been linked to hepatic necrosis, liver transplantation, and terminal multiorgan failure [2,8,9]. Although hepatocellular enzyme activities were not measured at the time of acute deterioration, the concurrent findings of severe hemolysis, marked hyperbilirubinemia, pigmenturia suggestive of hemoglobinuria, progressive azotemia with oliguria, hyperlactatemia, and systemic inflammation indicated severe systemic illness. Hypoxic, ischemic, inflammatory, and oxidative injury associated with severe hemolysis has been proposed as a possible contributor to lipopigment accumulation; however, these mechanisms were not directly evaluated in the present case and remain speculative.

The present case also differs temporally from the canine cases reported by Sebastian et al. [3]. In that study, 11 dogs with blue-green leukocyte inclusions were identified, all of which had biochemical evidence of hepatocellular injury, and the predominant clinical conditions included hepatic disease and pancreatitis. One dog received a pRBC transfusion and subsequently developed a transfusion reaction considered to have resulted in hemoglobinuric nephropathy; importantly, the lipofuscin-like inclusions had been detected before that transfusion reaction occurred. In contrast, in the present case, the inclusions were detected during the acute clinical deterioration following the second pRBC transfusion. Nevertheless, because no immediately pre-event smear was available and concurrent hepatocellular injury could not be excluded, this temporal difference does not establish transfusion-associated hemolysis as the cause of inclusion formation. Rather, the present case extends the clinical context in which morphologically and cytochemically comparable inclusions have been observed.

Several differential considerations should be recognized when interpreting the blue-green neutrophilic inclusions in this case. Hemosiderin-containing sideroleukocytes were particularly relevant given the suspected hemolytic episode; however, representative blue-green inclusions lacked distinct Prussian blue reactivity, reducing the likelihood of a predominantly hemosiderin-based explanation. Concurrent hepatocellular injury remains an important alternative because similar inclusions in dogs have been strongly associated with hepatocellular injury [3]. Severe systemic inflammation and critical illness may also have contributed to their occurrence. Thus, the available findings do not permit attribution of the inclusions to a single underlying process. Proposed mechanisms in previous reports, including uptake of cellular degradation products, lysosomal dysfunction, and accumulation of oxidized lipopigments, were not directly evaluated in this case and remain speculative. Although inclusion detection coincided with rapid clinical deterioration and death three days later, no independent diagnostic or prognostic significance can be inferred from a single case.

## 4. Limitations

This case report has several limitations. First, it represents a single case, limiting the ability to generalize the clinical significance of the observed inclusions. Second, archived blood smears obtained immediately before the acute post-transfusion deterioration were unavailable; therefore, it could not be determined whether the inclusions were absent before this event or developed during the subsequent clinical deterioration. Third, ALT and AST activities were not measured at the time of inclusion detection, and concurrent hepatocellular injury-a major previously reported association of blue-green leukocyte inclusions in dogs-could not be excluded. Fourth, the suspected transfusion-associated hemolytic event could not be definitively classified because post-reaction compatibility testing, direct antiglobulin testing, plasma-free hemoglobin measurement, formal post-reaction urinalysis, and detailed donor/unit information were unavailable. Fifth, no postmortem histopathologic examination was performed, limiting assessment of organ-level pathology and the potential origin of the inclusions. Finally, definitive biochemical or ultrastructural characterization of the inclusions was not performed; therefore, their identification as lipofuscin-like material remains based on combined cytomorphologic, cytochemical, and autofluorescence characteristics.

## 5. Conclusions

Blue-green cytoplasmic inclusions were identified in neutrophils of a dog during acute clinical deterioration temporally associated with suspected transfusion-associated hemolysis. Cytochemical staining and autofluorescence showed characteristics compatible with lipofuscin-like material, while the absence of distinct Prussian blue reactivity in representative inclusions reduced the likelihood of a predominantly hemosiderin-based composition; however, their definitive chemical identity was not established. Because immediately pre-event blood smears were unavailable and concurrent hepatocellular injury and other effects of critical illness could not be excluded, a causal relationship among transfusion, hemolysis, and inclusion formation cannot be established. This case expands the clinical context in which blue-green leukocyte cytoplasmic inclusions may be encountered in dogs.

## Figures and Tables

**Table 1:** Serial hematologic changes during the clinical course.

Clinical Phase	Relative Time Point	HCT (%) 37.3–61.7	RBC (10^12^/L) 5.65–8.87	HGB (g/dL) 13.1–20.5	MCV (fL) 61.6–73.5	MCH (pg) 21.2–25.9	MCHC (g/dL) 32–37.9	RDW (%) 13.6–21.7	Reti (K/μL) 10–110
Pre-1st transfusion	Day 0	7.7	1.07	2.3	72	21.5	29.9	ND	15.4
Post-1st transfusion	Day 2	37.4	6.75	13.7	55.4	20.3	36.6	18.6	35.1
Follow-up after 1st transfusion	Day 9	17.8	3.44	6.8	51.7	19.8	38.2	16.5	14.1
Follow-up after 1st transfusion	Day 12	16.9	3.23	6.5	52.3	20.1	38.5	16.7	10.0
Pre-2nd transfusion	Day 23	12.9	2.49	4.6	51.8	18.5	35.7	18.9	15.4
Immediately after 2nd transfusion	Day 23	36.4	5.97	12.0	61.0	20.1	33.0	17.3	17.3
Suspected transfusion-associated hemolysis	Day 24	11.4	2.34	7.1	48.7	30.3	62.3 *	21.9	21.5
Clinical deterioration during inclusion detection period	Day 24	9.8	1.95	5.7	50.3	29.2	58.2 *	17.6	37.1

* MCHC values > 37.9 g/dL reflect analytical interference caused by severe hemolysis and icterus, not true intracellular hemoglobin concentration.

**Table 2:** Serial biochemical changes during the clinical course.

Clinical Phase	Relative Time Point	ALT (U/L) 10–125	AST (U/L) 0–50	ALP (U/L) 23–212	GGT (U/L) 0–11	Total Bilirubin (mg/dL) 0–0.9	BUN (mg/dL) 7–27	Creatinine (mg/dL) 0.5–1.8	Lactate (mmol/L) 0.5–2.5
Pre-1st transfusion	Day 0	62	57	180	7	<0.1	17	0.5	6.81
Post-1st transfusion	Day 2	ND *	ND	ND	ND	ND	22	0.5	0.96
Follow-up after 1st transfusion	Day 9	ND	ND	ND	ND	ND	ND	ND	ND
Follow-up after 1st transfusion	Day 12	167	45	415	29	0.1	ND	ND	ND
Pre-2nd transfusion	Day 23	118	45	379	18	ND	ND	ND	ND
Immediately after 2nd transfusion	Day 23	ND	ND	ND	ND	ND	ND	ND	ND
Suspected transfusion-associated hemolysis	Day 24	ND	ND	ND	ND	17.9	65	1.0	4.02
Clinical deterioration during inclusion detection period	Day 24	ND	ND	ND	ND	ND	96	1.4	ND

* ND, not determined. Hepatocellular enzymes (ALT, AST) were not evaluated on Day 24 due to sample limitations during the acute hemolytic event.

## Data Availability

The original contributions presented in the study are included in the article and Supplementary Material. Further inquiries can be directed to the corresponding author.

## Supplementary Materials

The following supporting information can be downloaded at: https://www.mdpi.com/article/10.3390/vetsci13090985/s1, Supplementary material consists of the original uncropped images of Figure 2.

## Abbreviations

The following abbreviations are used in this manuscript:

HCT	Hematocrit
RBC	Red Blood Cell Count
HGB	Hemoglobin
MCV	Mean Corpuscular Volume
MCH	Mean Corpuscular Hemoglobin
MCHC	Mean Corpuscular Hemoglobin Concentration
RDW	Red cell Distribution Width
Reti-HGB	Reticulocyte Hemoglobin Content
ALT	Alanine Aminotransferase
AST	Aspartate Aminotransferase
ALP	Alkaline Phosphatase
GGT	Gamma-Glutamyltransferase
BUN	Blood Urea Nitrogen
CRP	C-Reactive Protein
ND	Not Determined
