# Supplementary material for: Cytologic and Cytochemical Characterization of Blue-Green Neutrophilic Inclusions in a Dog with Suspected Transfusion-Associated Hemolysis
Source: Vet Sci. 2026 Sep 18;13(9):985. doi: 10.3390/vetsci13090985 (PMC13612001; doi:10.3390/vetsci13090985)
Supplement: Supplementary file 1 [file vetsci-13-00985-s001.zip › vetsci-4544094-supplementary.pdf]

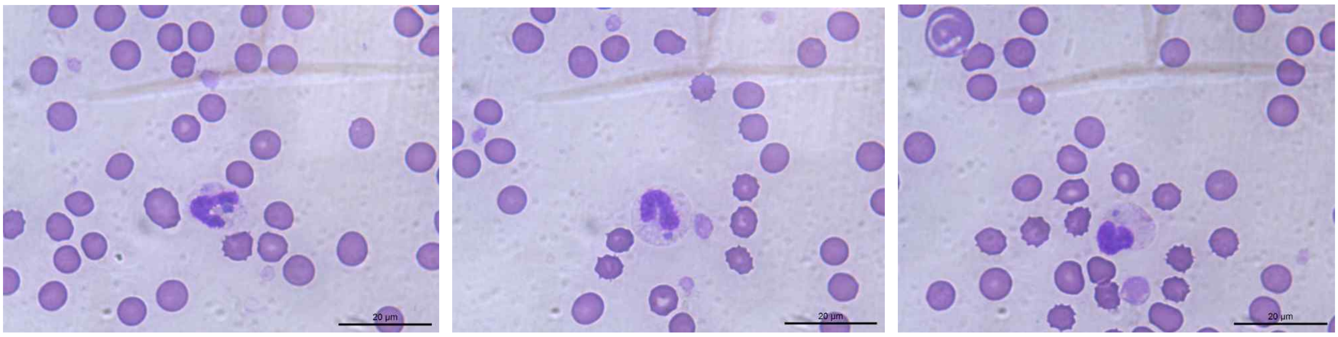

Figure 2A-2C. Uncropped Diff-Quik staining

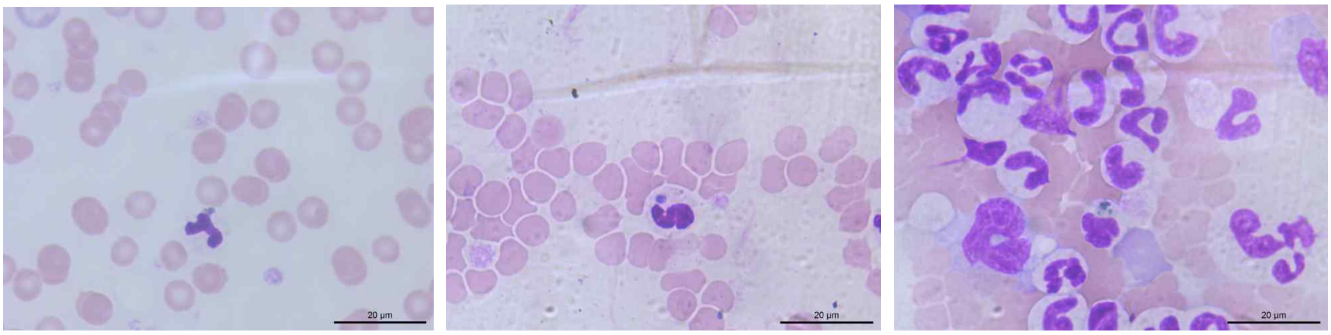

Figure 2D-2F. Uncropped Wright-Giemsa staining

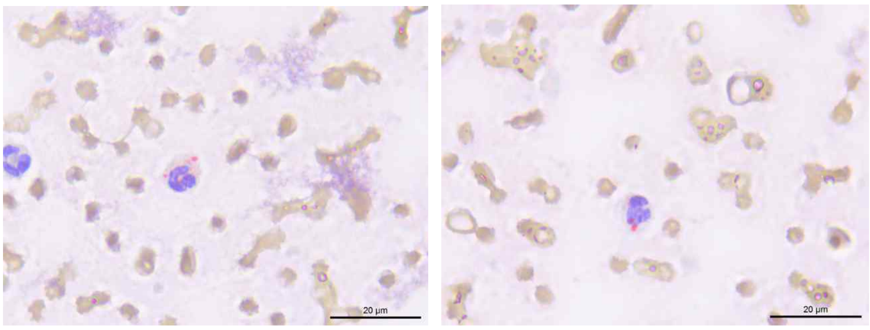

Figure 3A-3B. Uncropped Oil Red O staining

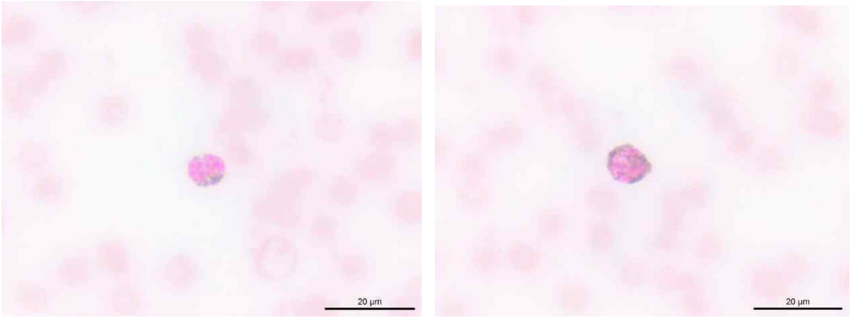

Figure 3C-3D. Uncropped Sudan Black B staining

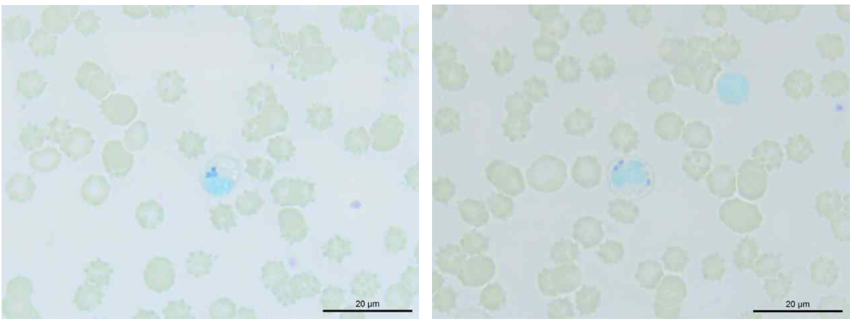

Figure 3E-3F. Uncropped Nile Blue staining

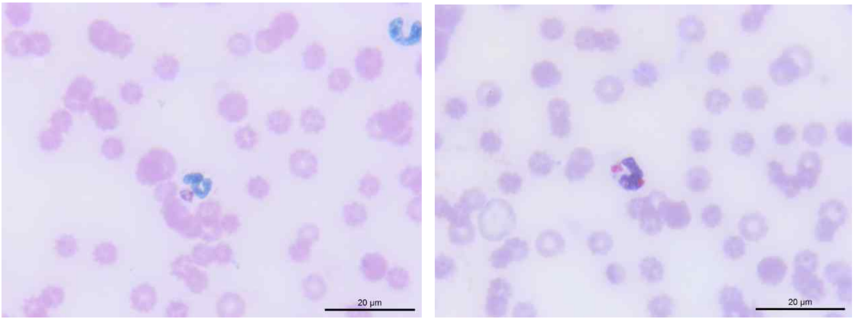

Figure 3G-3H. Uncropped Long Ziehl-Neelsen staining, counterstained with (3G) new methylene blue and (3H) hematoxylin

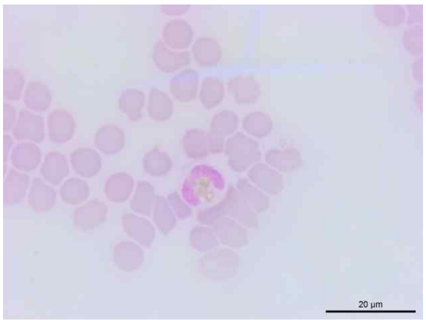

Figure 3I. Uncropped Prussian blue staining

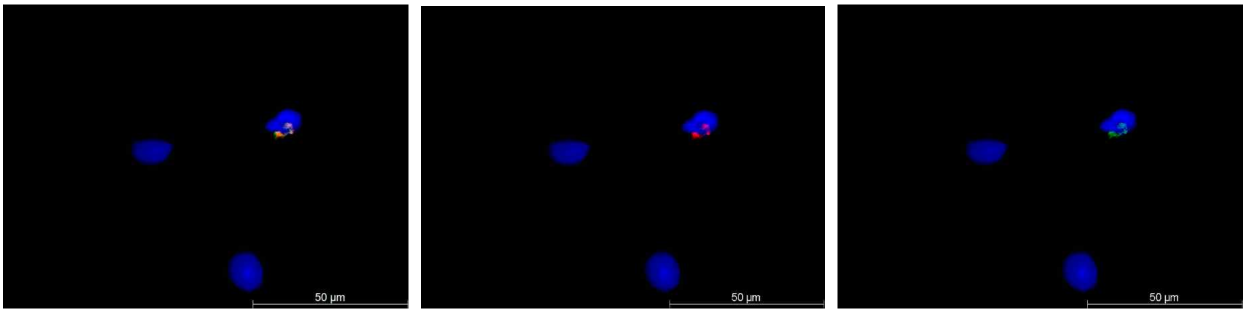

Figure 4. Uncropped (A) Merged images; (B) red fluorescence/DAPI merged; (C) green fluorescence/DAPI merged

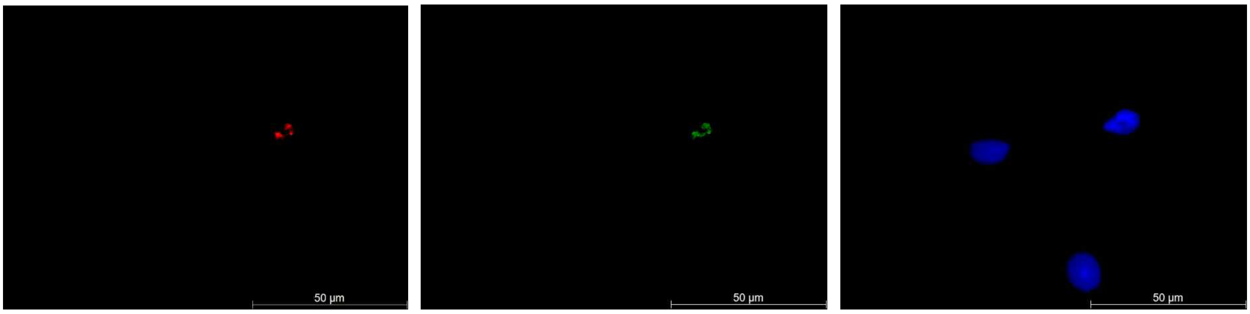

Figure 4. Uncropped (D) red fluorescence (N2.1); (E) green fluorescence (L5); (F) DAPI nuclear fluorescence (D)
